# Enhanced neural mechanisms of set shifting in musically trained adolescents and young adults: converging fMRI, EEG, and behavioral evidence

**DOI:** 10.1093/cercor/bhad034

**Published:** 2023-03-10

**Authors:** K Saarikivi, T M V Chan, M Huotilainen, M Tervaniemi, V Putkinen

**Affiliations:** Department of Education, Faculty of Educational Sciences, University of Helsinki, Siltavuorenpenger 5, Helsinki, 00170, Finland; Cognitive Brain Research Unit, Department of Psychology and Logopedics, Faculty of Medicine, University of Helsinki, Haartmaninkatu 8, Helsinki, 00290, Finland; Aalto NeuroImaging, Aalto University, Otakaari 5, Espoo, 02150, Finland; Department of Psychology, University of Toronto, 100 St. George Street, Toronto, ON M5S 3G3, Canada; Department of Psychology, University of Notre Dame, 390 Corbett Family Hall, Notre Dame, IN 46556, United States; Cognitive Brain Research Unit, Department of Psychology and Logopedics, Faculty of Medicine, University of Helsinki, Haartmaninkatu 8, Helsinki, 00290, Finland; Centre of Excellence in Music, Mind, Body, and Brain, Faculty of Educational Sciences, University of Helsinki, Siltavuorenpenger 5, Helsinki, 00170, Finland; Cognitive Brain Research Unit, Department of Psychology and Logopedics, Faculty of Medicine, University of Helsinki, Haartmaninkatu 8, Helsinki, 00290, Finland; Centre of Excellence in Music, Mind, Body, and Brain, Faculty of Educational Sciences, University of Helsinki, Siltavuorenpenger 5, Helsinki, 00170, Finland; Cognitive Brain Research Unit, Department of Psychology and Logopedics, Faculty of Medicine, University of Helsinki, Haartmaninkatu 8, Helsinki, 00290, Finland; Turku PET Center, University of Turku, Turku University Hospital, Kiinamyllynkatu 4-820520 Turku, Finland; Turku Institute for Advanced Studies, University of Turku, 20014 Turun yliopisto, Turku, Finland; Turku University Hospital, Turku, Finland

**Keywords:** dorsal attention network, ERP, musical training, P3b, set shifting

## Abstract

Musically trained individuals have been found to outperform untrained peers in various tasks for executive functions. Here, we present longitudinal behavioral results and cross-sectional, event-related potential (ERP), and fMRI results on the maturation of executive functions in musically trained and untrained children and adolescents. The results indicate that in school-age, the musically trained children performed faster in a test for set shifting, but by late adolescence, these group differences had virtually disappeared. However, in the fMRI experiment, the musically trained adolescents showed less activity in frontal, parietal, and occipital areas of the dorsal attention network and the cerebellum during the set-shifting task than untrained peers. Also, the P3b responses of musically trained participants to incongruent target stimuli in a task for set shifting showed a more posterior scalp distribution than control group participants’ responses. Together these results suggest that the musician advantage in executive functions is more pronounced at an earlier age than in late adolescence. However, it is still reflected as more efficient recruitment of neural resources in set-shifting tasks, and distinct scalp topography of ERPs related to updating and working memory after childhood.

## Introduction

Executive functions are control mechanisms that enable self-regulation and higher order phenomena, such as complex problem solving, goal-directed behavior, planning, and reasoning (reviews: e.g. Stuss and Alexander 2000; Jurado and Rosselli 2007; Diamond 2013). Behavioral studies have uncovered 3 correlated but separable core components of executive functions: inhibition, cognitive flexibility, and working memory (Miyake et al. 2000; Lehto et al. 2003; Miyake and Friedman 2012; Diamond 2013; Herd et al. 2014; Friedman and Miyake 2017; Karr et al. 2018). Cognitive flexibility is further divided into fluency, or the ability to relinquish inhibition and generate information from the mind, and *set shifting, or task-switching*, the abilities to fluidly change response strategies within a task, or fluidly change between 2 tasks. Executive functions rely on the prefrontal cortex and their diverse connections to other brain regions (Alvarez and Emory 2006; Yuan and Raz 2014). These include the “dorsal attention network,” consisting of the dorsolateral prefrontal cortex, frontal eye fields, inferior precentral sulcus, and the superior parietal lobule (Fox et al. 2005; Toro et al. 2008) and the “frontoparietal control network” comprising the rostrolateral prefrontal cortex, middle frontal gyrus, anterior insula, dorsal anterior cingulate cortex, precuneus, and anterior inferior parietal lobule (Corbetta and Shulman 2002; Badre and D’Esposito 2007; Vincent et al. 2008; Spreng et al. 2010; Szczepanski et al. 2013). There is also evidence of involvement of subcortical structures in executive functions, including the cerebellum (Brissenden et al. 2016; Ramanoel et al. 2018; Habas 2021) and the basal ganglia (Hazy et al. 2007; Saban et al. 2018).

The subcomponents of executive functions are distinguishable on the neural level. fMRI studies have shown that the frontoparietal control network exhibits different patterns of connectivity for working memory and inhibitory control (Harding et al. 2015). Cognitive flexibility and working memory have in turn been connected to 2 distinct networks, the fronto-parietal and the cingulo-opercular, respectively (Dosenbach et al. 2008; Cole et al. 2013; Power and Petersen 2013). Inhibition has been connected to right-lateralized activation of the dorsolateral prefrontal cortex (Zheng et al. 2008), the frontal inferior gyrus, and the pre-supplementary motor area (Sharp et al. 2010), reflecting the attention and motor processes involved in inhibiting prepotent responses.

In EEG studies, P3b responses emerge to target stimuli in different tasks for executive functions, and have been found to predict performance in these tasks. Recently, the amplitude of P3b responses to to-be-remembered stimuli in a working memory task was connected to working memory capacity (Lenartowicz et al. 2021). In task switching experiments, larger P3b-like response amplitudes have been recorded to targets during switching than updating trials, and to targets presented immediately after a switching cue compared to repeating trials (Barcelo et al. 2018; Brydges and Barceló 2018).

Some executive functions are already present at birth, and they mature rapidly during preschool age (Garon et al. 2008). However, the developmental trajectory of executive functions is prolonged, extending to school-age and adolescence, and coinciding with the development of particularly the prefrontal cortex but also other brain areas associated with executive functions, such as the anterior cingulate cortex and basal ganglia (Blakemore and Choudhury 2006; Best and Miller 2010). The mechanisms of development could include synaptic pruning, which takes place last in prefrontal areas, myelination, and thickening or thinning of the cortex (Best and Miller 2010).

Musically trained individuals have been found to outperform untrained peers in tasks of inhibition and set shifting (Bialystok and DePape 2009; Bugos and Mostafa 2011; Degé et al. 2011; Moreno et al. 2011; Zuk et al. 2014; Saarikivi et al. 2016, 2019; Strong and Mast 2019). Longitudinal studies with children undergoing music training, and studies on music training interventions, have found training-related improvements in working memory (Roden et al. 2013; Nie et al. 2021; Frischen et al. 2021) and inhibition (Moreno et al. 2011; Bugos and DeMarie 2017; Jaschke et al. 2018; Frischen et al. 2019; Frischen et al. 2021), pointing towards a causal role for music training in the enhancement of executive functions (however, see Bialystok and DePape 2009; Schellenberg 2011; Janus et al. 2016; Linnavalli et al. 2018). Music training requires executive functions in many ways that are not restricted to the auditory domain. For instance, inhibitory control over distracting sounds is needed when attending to one’s own instrument in joint playing, and working memory is required for playing melodies by ear. Set shifting is an integral part of playing from notes as the musician transforms visual stimuli into motor responses, shifting between response strategies based on sharp (♯), flat (♭), and other symbols.

Neuroimaging and electrophysiological studies have also reported differences between musically trained and untrained individuals in indices of executive functions. For instance, adult musicians show larger P3a responses than nonmusicians in oddball paradigms (Trainor et al. 1999; Brattico et al. 2009; Vuust et al. 2009; Virtala et al. 2018), indicating faster attention switching towards sound changes. Adult musicians have also been found to exhibit larger P3b responses than nonmusicians to deviant sounds, signaling more efficient updating and working memory processing (Trainor et al. 1999; Carrión and Bly 2008; Nikjeh et al. 2009). Longitudinal studies with children engaged in musical activities have also reported enhancement of P3as recorded in an oddball paradigm, signaling better deviance processing, smaller P3as elicited by novel sounds, signaling decreased distractibility (Putkinen et al. 2014, 2021), and larger P2 responses to no-go targets during a go/no-go task, signaling better inhibitory control (Moreno et al. 2014).

fMRI studies have revealed both increased and decreased activation in brain areas related to executive functions in musically trained individuals compared to untrained peers. Pallesen et al. (2010) found stronger activation with increasing working memory load in adult musicians relative to non-musicians in various regions linked with executive functions including the right frontal and parietal cortices, right insula, and the cingulate. Similarly, Kausel et al. (2020) found that musically trained 10–13-year-old children showed higher activation than their untrained peers in the fronto-parietal control network during encoding in a working memory task. In addition, better performance in the task correlated with years of training and higher activity in the left inferior frontal gyrus and the left supramarginal gyrus (SMG), related to working memory. In a longitudinal study with older adults (Guo et al. 2021), a 4-month music training intervention increased activation in the right supplementary motor area and left precuneus, but decreased activation in the bilateral posterior cingulate gyrus as well as connectivity between the right posterior cingulate gyrus and the left middle temporal gyrus, and between the left putamen and the right superior temporal gyrus during the forward and backward digit span tasks, used to assess working memory task. Here, larger improvements in the working memory task were associated with larger reductions in connectivity between the aforementioned regions. Similarly, in Alain et al. (2018), adult musicians showed less activation in the prefrontal cortex bilaterally than non-musicians when performing an auditory N-back working memory task. Here, participants listened to a stream of stimuli and were required to indicate whether an item presented matched an item that had been presented a variable number (*n*) of items back.

Differences between musically trained and untrained children have also been uncovered in brain activations related to inhibition and set shifting. In their study, Sachs et al. (2017) found that children 8–9 years of age attending music training had stronger bilateral activation of the pre-supplementary motor area, anterior cingulate cortex, the inferior frontal gyrus (IFG), and the insula during a Stroop task when compared to control group participants, but not an active control group taking part in sports. In a study with the same participants, 4 years later, these differences were only present in the right IFG (Hennessy et al. 2019). A study by Zuk et al. (2014) in turn found that 9–12-year-old musically trained children recruited the supplemental motor area and ventrolateral prefrontal cortex more strongly than untrained children in a set-shifting task. However, the study was underpowered due to the small sample size (total *n* = 27 in groups of 15 musically trained vs. 12 untrained children). Further, the above-mentioned group differences were only seen with an uncorrected threshold, while more subtle differences between the groups might have been missed even with the liberal threshold. In sum, a view on differences between musically trained children and adolescents in brain activity related to particularly set shifting is still missing.

Together, behavioral and neuroimaging studies point towards the potential of music training in augmenting the development of executive functions. However, studies extending from childhood to adolescence are rare, and consequently the age-related trajectory of possible music-training-related enhancement of executive functions remains unclear. Furthermore, the neural mechanisms of particularly the enhancement of set shifting in musically trained individuals are understudied. It is possible that in late adolescence and early adulthood, the development of the most demanding executive functions such as set shifting is at its peak, and possible benefits gained from musical training in simple set-shifting tasks will begin to dissipate when comparing behavior between trained and untrained individuals.

In this study, we set out to fill these gaps by investigating possible connections between music training and the maturation of the mechanisms of set shifting during late adolescence and early adulthood. To this end, we measured behavioral performance, P3b event-related potentials (ERPs) to target stimuli, and BOLD activations in a task for set shifting in musically trained and untrained adolescents aged 13–21 years. To build a better understanding of how far into adolescence possible enhancement of set shifting persists, we combined results from the neuropsychological tests from 2 previous measurement rounds, obtained 2–4 years prior to the brain measurements. We expected more efficient recruitment of areas related to executive functions, such as the dorsal attention network, in musically trained individuals compared to untrained peers during set-shifting task trials, and that P3b responses to incongruent stimuli in the EEG version of the task would be larger in amplitude in the music group than in the control group. Finally, we expected an age-related decrease in differences in a task for set shifting.

**Table 1:** Means ages and numbers of participants in the music and control groups for the ERP, fMRI experiment, and neuropsychological tests.

	**ERP**		**fMRI**		**Tests**	
	**Music**	**Control**	**Music**	**Control**	**Music**	**Control**
Mean age (SD, range)	17.03 ± 2.36	17.22 ± 2.42	18.3 ± 1.66	18.3 ± 1.72	17 ± 2.32	17.1 ± 2.4
*N*	35 (9 male)	28 (16 male)	23 (7 male)	21 (11 male)	51 (20 male)	52 (27 male)

## Methods

### Participants

The participants are from a 14-year longitudinal study called the Early Auditory Skills project, conducted at the University of Helsinki, mapping neurocognitive development in musically trained and untrained children and adolescents. Seven-year-olds were invited to participate in measurements every 2 years, and a new group of children was recruited on each measurement round. Therefore, the participants who were recruited earlier took part in several measurements while those recruited later had fewer measurements. The last measurements included in the longitudinal study were conducted in 2017, and altogether, there were 7 measurement rounds.

The longitudinal study first employed experiments recording auditory ERPs. For the last 3 measurement rounds, neuropsychological tests for executive functions were also included, and on the last round, the fMRI and ERP experiments reported here.

On average, the participants of this study were measured with neuropsychological tests 2.43 times. The fMRI and EEG data reported here were collected only once, during the last measurement round of the longitudinal study.

The participants in the music group had played an instrument starting at age 7 and attended a specialized public elementary school that included classical music instruction in the regular curriculum. The untrained participants were recruited from regular elementary schools, have no formal music training, and match the music group participants in age, IQ as measured with the Vocabulary and Block design subtests from the fourth edition of the Wechsler Intelligence Scale for Children (WISC-IV), and socioeconomic status as measured by parental income and education (Income scale: 1 = under a 1,000 Euros/month, 2 = 1,000–2,000 Euros/month, 3 = 2,000–3,000/month, 4 = 3,000–4,000/month, 5 = 4,000–5,000 Euros/month, 6 = over 5,000/month; Education scale: 1 = comprehensive school, 2 = upper secondary school or vocational school, 3 = a higher degree than upper secondary school or vocational school, 4 = Bachelor’s degree or equivalent, 5 = Master’s degree or equivalent, 6 = licentiate or doctoral-level degree) (Putkinen et al. 2014; Saarikivi et al. 2016). Most participants in both groups were from middle-class and upper-middle-class families from the Helsinki metropolitan area, and no participants had neurological, hearing, or sight disturbances.

Forty-four participants (23 musically trained) aged 16–21 took part in the fMRI experiment, 63 (35 musically trained), aged 13–21 in the EEG experiment, and 103 (51 musically trained) completed the neuropsychological tests. Data from neuropsychological tests from 2 previous measurement rounds were available for 24 participants and from 1 previous measurement round for 20 participants.

Table 1 details the mean ages and number of participants in each experiment.

### Procedure

During the EEG experiments, the participants sat in an armchair in an acoustically attenuated and electrically shielded room. Visual stimuli were presented on a computer screen placed 1.5 m in front of the participant. The neuropsychological set-shifting task was administered in another quiet room by a psychology major before the EEG experiment. An experimental session with EEG and neuropsychological tests lasted for ~2.5 h. Tests took ~45 min, the EEG recording about 60 min, with 45 min reserved for discussion with the participants about the procedure, breaks, and for placing and removing the electrode cap. The MRI and fMRI acquisitions were conducted on a separate occasion after the EEG and neuropsychological test at the AMI Centre of the Aalto University School of Science.

The required safety screening measures were administered prior to acquisition. Especially with the youngest participants, here 16–17-year-olds, special care was taken to ensure comfort, for instance by monitoring the emotional state of the participants and reacting with assurance to possible distress, and by ensuring that participants had enough knowledge of what was going on to create a sense of predictability and control.

Signed informed consent was obtained before measurements from the parents of all underaged participants. A separate signed consent was also obtained from 16 to 17-year-olds and oral consent was obtained from participants of all ages before each measurement session (EEG and fMRI). Separate information sheets were designed for participants of different ages to ensure that oral or written consent was truly informed, and that the procedure was explained in an age-appropriate way. Before starting the EEG and fMRI measurements, the participants performed a short practice run, requiring the participant to respond to the arrow stimuli with the appropriate responses, to ensure that the task was understood before commencing the actual task. Measurements were conducted by psychologists, or students of psychology or cognitive science, who took special care to monitor the state of young participants, inform the participants about what is going on, and ensure that the measurements were as comfortable as possible. The participants were awarded movie tickets for their efforts. The experiment protocol for all measurements was approved by the Ethical Committee of the former Department of Psychology, University of Helsinki, Finland.

### E‌EG recording and MRI/fMRI acquisition

The BioSemi Active-Two system was used with a sampling rate of 512 Hz, and 64 Ag–AgCl scalp electrodes mounted in a BioSemi head cap according to the International 10–20 system. Three additional active electrodes were placed on the nose and on the right and left mastoid to act as reference channels (only the mastoids were used in analyses). Electro-oculogram for later removal of blinks and eye movements was recorded with electrodes placed above and at the outer canthus of the right eye.

The MRI/fMRI images were acquired with a 3-T MAGNETOM Skyra whole-body scanner (Siemens Healthcare, Erlangen, Germany) with a 20-channel head coil. Functional echo planar images (EPI) were acquired with an imaging area consisting of 42 contiguous oblique axial slices with the following parameters: TR 2,500 ms, TE 32 ms, flip angle 75°, voxel matrix 64 × 64, field of view 19.2 cm, in-plane resolution of 3 × 3 mm, and slice thickness of 3 mm. Image acquisition was performed at a constant rate, but it was asynchronized with stimulus onsets. Structural images were acquired using a T1-weighted sequence (3D MP-RAGE) with a resolution of 1 mm^3^, using a 256 × 256 voxel matrix.

### Neuropsychological tests

The inhibition test from the NEPSY-II test battery (Korkman et al. 2008) was administered to investigate set shifting. Even though the test is named inhibition it in fact contains 3 tasks: naming, inhibition, and set shifting. In the first task, the participant simply names shapes (square/circle) and the directions of arrows (up/down). The second task requires the participant to name the opposite shape (“circle” if square; “square” if circle) and the direction of the arrow (“up” if down; “down” if up). The third task, set shifting, requires the participant to switch between naming the shape or the direction of the arrow and naming the opposite shape or direction, based on the color of the shape or the arrow (if white, naming; if black, inhibition and naming the opposite). The shapes and arrows are presented to participants from a booklet placed on a table in front of the participant. The participant may point to the shapes or arrows on the booklet while reciting answers. The number of errors and completion time are used as performance indexes. Only performance in the third task, set shifting, was analyzed in this study.

### The EEG and fMRI paradigms

fMRI and EEG versions of the set-shifting task of the inhibition test from NEPSY-II were created to acquire Blood Oxygen Level-Dependent (BOLD) activations and ERPs related to task performance. In transforming the task to suit neuroimaging settings, and to allow the capture of shifting-related brain activity, alterations were made. In comparison to the neuropsychological set-shifting task, the fMRI and EEG versions required a motor instead of verbal response to reduce movement artifacts caused by speech. Also the pacing of the tasks used in neuroimaging differed from the original task. In the fMRI and EEG versions, obtaining event-related activations required a task where the responses could be obtained within a specific timeframe of sufficient duration, and not according to the participant’s own pace as in the original task. Lastly, in the fMRI and EEG versions, reaction times (RTs) were used as an index of performance instead of total completion time of the task, as in the neuropsychological set-shifting task.

The Arrows fMRI paradigm had 4 naming, 4 inhibition, and 8 set-shifting task blocks presented in random order. While in the scanner, the participant viewed a white or black arrow at the center of the screen pointing to the left or right and was asked to press a button with the left or right hand according to the instructions shown on the screen at the beginning of the block. In baseline or naming blocks, the instruction was to press the button in the same direction that the arrow was pointing, and in the inhibition blocks, the task was to respond in the opposite direction. In the set-shifting blocks, the participant was instructed to respond with the congruent or incongruent hand depending on the color of the arrow. This means that the participant had to switch between response strategies (naming and inhibition) when the color of the arrow changed. The rule was also changed so that on different runs, the black color signified naming, and white inhibition, and vice versa. Thus, there was no consistent mapping between the color of the arrow and the required response throughout the experiment and the participant had to keep in mind the rule that was presented on screen before the run. The switch between rules during the task required shifting between 2 different response rules in mind, and was designed to elicit brain activity specific to shifting. Fifty percent of the trials in the set-shifting blocks required the incongruent response and the other half the congruent response, requiring switching between the congruent trials that correspond to the naming condition and the incongruent trials that correspond to the inhibition condition.

Our main interest was to investigate how the additional shifting and working memory demands in the set-shifting condition would affect the contrast between the shifting-congruent vs. baseline and shifting-incongruent vs. inhibition contrasts and how brain activity revealed by these contrasts differ between the groups.

**Fig. 1:**
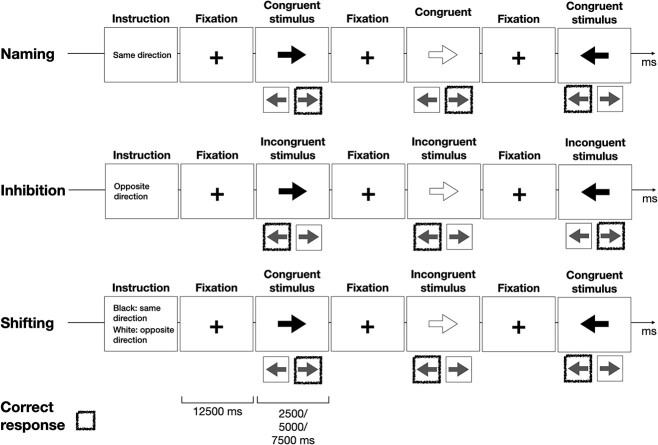
Illustration of the Arrows fMRIparadigm. A sequence of black and white arrows was presented with the task of pressing a button in the same or in the opposite direction, either irrespective of (naming and inhibition) or depending on (set shifting) the color of the arrow, according to the instruction of the set. The instruction for the set-shifting set was as depicted for 4 blocks and reversed (black, different direction) for the other 4 blocks. BOLD responses to congruent and incongruent stimuli and response times were included in analyses.

The arrow stimuli in the Arrows fMRI paradigm were presented for 2,500, 5,000, or 7,500 ms. A black fixation cross on a white background was presented between trials for 12.5 s. Forty eight trials for each condition (baseline, inhibition, set shifting) were presented, for a total of 192 trials, which were split over 2 runs. The shifting rule, i.e. the color of the arrow indicating whether to respond with a congruent or incongruent button press, was switched between runs. Fig. 1 shows examples of the baseline, inhibition, and set-shifting trials along with correct responses.

**Fig. 2:**
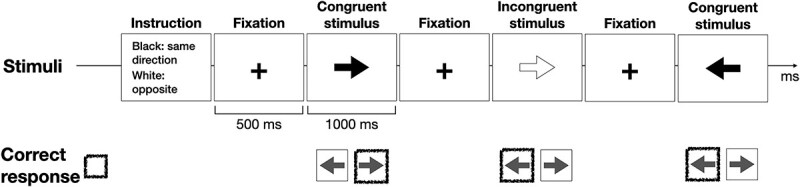
Illustration of the Arrows EEGparadigm. A sequence of black and white arrows was presented with the task of pressing a button in the same or in the opposite direction, depending on the coloration of the target arrow. ERPs to congruent and incongruent stimuli and response times were included in analyses.

The Arrows EEG paradigm (Fig. 2) included only a set-shifting condition, requiring the participant to switch between a congruent and an incongruent response according to the color (black or white) of the stimulus. The stimuli were presented for 1,000 ms, and the stimulus onset asynchrony was 1,500 ms. There were altogether 100 stimulus presentations per run, and 2 runs. Here too, the shifting rule changed between runs. The stimuli were presented in pseudorandom order with no more than 2 of the same stimuli in sequence. The total duration of the task was 2 × 2.5 min.

### Data analysis

#### fMRI preprocessing and analyses

fMRI data preprocessing was carried out using the fMRIprep pipeline (Esteban et al. 2019). Structural T1-weighted (T1w) images were corrected with N4BiasFieldCorrection (Tustison et al. 2010). Brain surfaces were reconstructed using recon-all (FreeSurfer 6.0.1, Dale et al. 1999). Brain tissue segmentation was performed on the brain-extracted T1w using fast (FSL 5.0.9, Zhang et al. 2001). Functional data were slice-time corrected using 3dTshift from AFNI 20160207 (Cox and Hyde 1997). The BOLD time-series were resampled and corrected for head motion. The BOLD reference was co-registered to the T1w using bbregister (FreeSurfer). Finally, functional data were spatially smoothed with a Gaussian kernel (9.0 mm FWHM).

Analyses of fMRI data were conducted with SPM12. To reveal regions activated by naming, inhibition, and set shifting, a general linear model (GLM) was fitted to the data where the design matrix included regressors for the stimuli in each condition (i.e. 4 regressors altogether). The following contrast images were computed: set-shifting trials > inhibition and naming trials, incongruent set-shifting trials > inhibition trials, and congruent set-shifting trials > inhibition trials. The effects of group were modeled in the second-level analysis with age and sex as covariates. Clusters surviving family-wise error (FWE) correction (*P* < 0.05) are reported.

#### E‌EG preprocessing

EEG data preprocessing and analyses were conducted in MATLAB using the EEGLAB toolbox (v. 13.5.4b; Delorme and Makeig 2004). The data were re-referenced to the average of the mastoid channels, and bad channels were excluded, and channel drift removed with the clean_rawdata plugin. Flatline criterion was set to lack of activity for 5 s or more, minimum acceptable correlation to other channels 0.8, and a filter transition band of 0.25–0.75 Hz was used to remove channel drift. Independent component analysis (ICA) was conducted and the ICLabel plugin was used to identify and then remove artefactual components such as eye blinks. The clean_rawdata plugin was then used to employ artifact subspace reconstruction (ASR, Kothe and Makeig 2013) to clear the data of any remaining artifacts. The standard deviation cutoff for removal of bursts (via ASR) was set to 20, and the parts of data where the variance was larger were removed. The data were then low pass filtered at 30 Hz, and epochs from 100 ms before to 1,500 ms after stimulus onset were extracted. Bad channels were interpolated. After this, epochs were averaged separately for congruent and incongruent stimuli.

**Table 2:** Results of the ANOVA on the effects of group and stimulus type (congruent vs. incongruent) on RTs in the Arrows EEG task and the Arrows fMRI task.

	**Effects**	**DFn**	***F*-value**	***P*-value**	**Ges**
**Arrows EEG**					
	Group	1	2.22	0.141	0.034
	Stimulus	3	32.4	**0.000** ^***^	0.023
	Group × Stimulus	3	0.68	0.414	0.000
**Arrows fMRI**					
	Group	1	0.03	0.872	0.001
	Stimulus	3	189.11	**0.000** ^***^	0.455
	Group × Stimulus	3	0.71	0.548	0.003

Ges = Generalized eta squared; DFn = Degrees of Freedom.

^***^ *P* ≤ 0.001.

**Table 3:** Results of the ANOVA on the effects of group and stimulus type (congruent vs. incongruent) on the number of correct responses in the Arrows EEG task and the Arrows fMRI task.

	**Effects**	**DFn**	***F*-value**	***P*-value**	**Ges**
**Arrows EEG**					
	Group	1	1.58	0.214	0.022
	Stimulus	3	2.69	0.106	0.005
	Group × Stimulus	3	1.48	0.229	0.003
**Arrows fMRI**					
	Group	1	0. 54	0.468	0.007
	Stimulus	3	25.12	**0.000** ^***^	0.204
	Group × Stimulus	3	0.04	0.988	0.000

Ges = Generalized eta squared.

^***^ *P* ≤ 0.001.

#### Behavioral performance and P3b responses in the Arrows EEG task

Differences in performance as measured with RTs and the number of incorrect responses in the Arrows EEG and the Arrows fMRI task were modeled with ANOVA (Group (Music vs. Control) × Stimulus (incongruent vs. congruent)).

To further study the effects of group, age, and channel location on the P3b, mean amplitudes calculated over a 50-ms time window centered at 350 ms at electrodes FC3, FCz, FC4, P3, Pz, P4, Oz, and O2 were subjected to a repeated measures ANOVA with factors Group (Music vs. Control) × Stimulus (incongruent vs. congruent) × Left-Center-Right × Anterior–Posterior (frontal-central-posterior). To quantify the connections between response amplitude and task performance, correlations between the response amplitude at POz (where the response was maximal) and RTs were computed separately for the incongruent and congruent trials.

#### Longitudinal performance in the neuropsychological set-shifting task

The effect of age and group membership on set-shifting test performance across 3 measurement rounds was modeled with a linear mixed model using the lmer function with Satterthwaite approximation for degrees of freedom of the lme4 package in R (Bates 2005; Bates et al. 2007). Age was mean-centered so that the significant effect of Group indicates a group difference in the test score at the average age of the participants. Linear mixed modeling was selected as the analysis since it allows different numbers of data points across subjects and accounts for correlations of the data within a subject.

## Results

### Behavioral results

In both the Arrows EEG and Arrows fMRI tasks, ANOVA revealed longer RTs for the incongruent than congruent stimuli in both the music and control groups (main effect of stimulus). No significant group differences were found in RTs in either the EEG or the fMRI versions. The average RT in the Arrows EEG in the music group was 552 ms, SD 61 ms, and in the control group 532 ms, SD 47 ms. Also, no significant effects of group membership emerged on the number of correct responses in either the Arrows EEG or the Arrows fMRI tasks. The results of the ANOVAs are summarized in Tables 2 and 3.

**Fig. 3:**
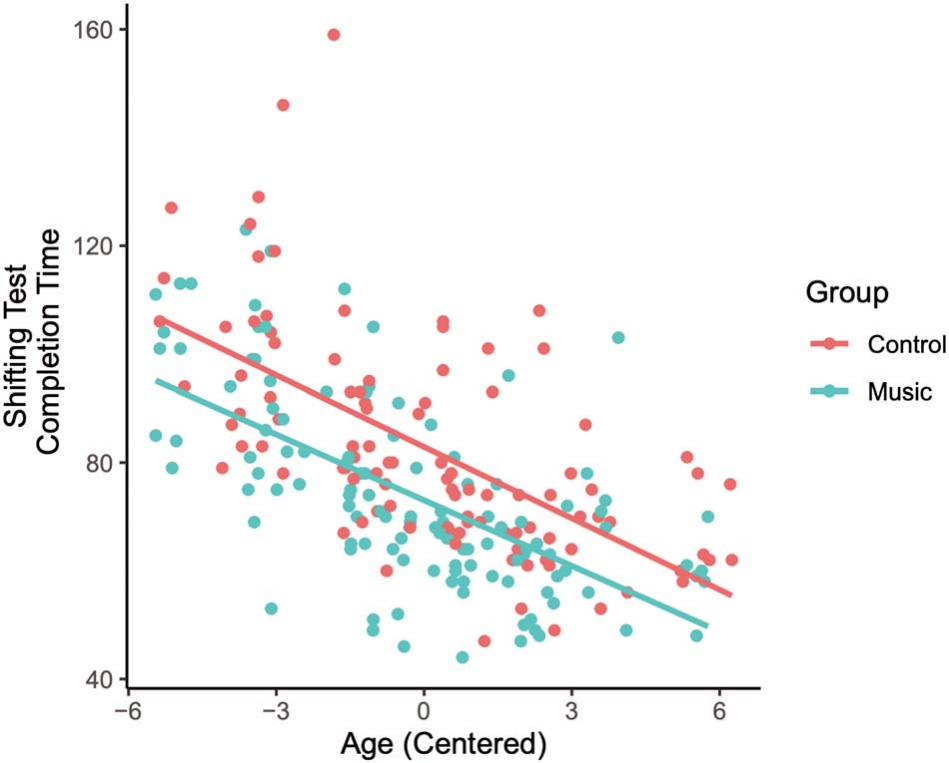
Set-shifting completion time as a function of mean-centered age. A significant group (music vs. control) difference in set-shifting test performance emerged.

The linear mixed model on the effects of age and group on performance in the neuropsychological test for set shifting revealed significant main effects of age and group, but no significant interaction, summarized in Table 4. Fig. 3 shows the test performance in the 2 groups as a function of mean-centered age.

**Table 4:** Results of the linear mixed model analysis on the effects of age and group membership on test performance.

Effects	Mean sq	NumDF	DenDF	*F*-value	*P*-value
Age	20054.1	1	211	156.97	**0.000** ^***^
Group	1885.1	1	100	14.76	**0.000** ^***^
Age × Group	53.0	1	209	0.42	0.520

NumDF = Numerator degrees of freedom; DenDF = Denominator degrees of freedom.

^***^ *P* ≤ 0.001.

Even though there was no interaction between age and group, we still tested whether data from only the last measurement round with the oldest participants, aged 13–21, would show an effect of music training, as the behavioral results of the Arrows EEG task showed no effect of group. A Welch 2 sample *t*-test revealed no group differences in the set-shifting task in this subgroup (*t* = 1.3031, df = 57.368, *P* = 0.1978).

### E‌EG results

**Fig. 4:**
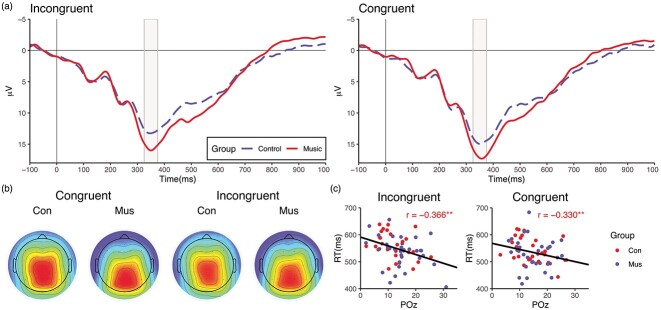
P3b responses at POz (a) and scalp topographies of the P3b response (b) to congruent and incongruent stimuli, separately for the music and control groups. Correlations between P3b amplitude and RTs in the Arrows EEG task (c). Time 0 = stimulus onset.

Both the incongruent and congruent stimuli in the Arrows EEG paradigm elicited a parietally maximal P3b-like response (Fig. 4a and b). RTs in the task correlated negatively with the P3b amplitude, i.e. the larger the response, the faster the RT, irrespective of age or group membership (Fig. 4c). There were no significant correlations between response latency and RTs for the congruent (*r* = 0.108, *P* = 0.399) and incongruent (*r* = 0.118, *P* = 0.357) stimuli.

ANOVA on group differences in topography indicated that the P3b was more posteriorly distributed in the music group (*P* = 0.0208, with Greenhouse–Geisser correction *P* = 0.040). Table 5 summarizes the results of the ANOVA.

**Table 5:** Results of the ANOVA on the effects of age, group membership, stimulus, and location on response amplitude.

**Effects**	**DFn**	**DFd**	***F***	***P***
Group	1	61	0.8066537	0.3730
Stimulus	1	61	13.5613599	0.0005^**^
Left-center-right	2	122	28.6353835	0.0000^***^
Anterior/posterior	2	122	37.3381931	0.0000^***^
Group × stimulus	1	61	0.6259287	0.4319
Group × left-center-right	2	122	0.3055774	0.7373
Group × anterior/posterior	2	122	3.9991336	0.0208^*^
Stimulus × left-center-right	2	122	3.6581879	0.0286^*^
Stimulus × anterior/posterior	2	122	3.7692873	0.0258^*^
Lat × anterior/posterior	3	244	34.9482195	0.0000^***^
Group × stimulus × left-center-right	2	122	2.8375499	0.0624
Group × stimulus × anterior/posterior	2	122	0.8888194	0.4138
Group × left-center-right × anterior/posterior	4	244	1.8379597	0.1222
Stimulus × left-center-right × anterior/posterior	4	244	1.7728853	0.1349
Group × stimulus × left-center-right × anterior/posterior	4	244	1.0818291	0.3661

^*^ *P* = <0.05 ^**^P = <0.01, ^***^P = <0.001.

### fMRI results

**Fig. 5:**
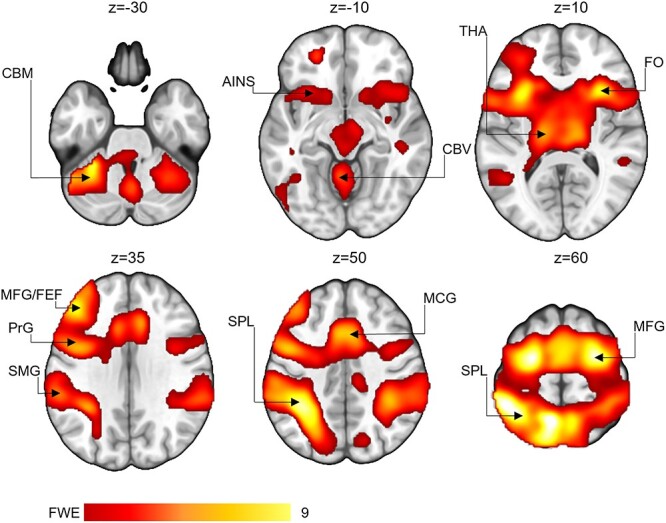
Activation during the set-shifting condition when contrasted with the naming and inhibition conditions. Activations in frontal (FO: frontal operculum, MFG/FEF: middle frontal gyrus, frontal eye field, MCG: middle central gyrus), temporal (SMG: supramarginal gyrus, PrG: precentral gyrus), and parietal (SPL: superior parietal lobule) cortices, the anterior insula (AINS), the thalamus (THA), and the cerebellum (CBM: cerebellum, CBV: cerebellar vermis) across groups. The color bar indicates *t*-value.

The fMRI data indicated that the set-shifting condition (all set-shifting trials > naming and inhibition conditions) activated areas of the frontal, temporal and parietal cortices, the anterior insula, and the cerebellum (Fig. 5).

**Fig. 6:**
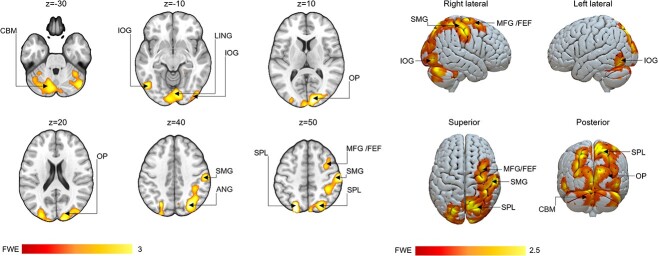
The control group showed higher activity (*P* < 0.05, corrected for multiple comparisons at the cluster level) than the music group for the set-shifting incongruent inhibition contrast in areas of the dorsal attention network including the right SMG and middle frontal gyrus/the frontal eye field (MFG/FEF), the superior parietal lobules (SPL), the angular gyrus (ANG), the lingual gyrus (LING) as well as the cerebellum (CBM), and in areas of the visual cortex (OP: occipital poles, IOG: inferior occipital gyri). The color bar indicates *t*-value.

With regard to group differences, the contrast between the incongruent trials in the set-shifting condition and the inhibition trials (i.e. identical stimuli and stimulus–response mapping) revealed stronger right-lateralized activity in the control group than in the music group in areas of the dorsal attention network (Fig. 6) including the right SMG and middle frontal gyrus/frontal eye field, the superior parietal lobules, the angular gyrus, the lingual gyrus, the cerebellum, as well as the occipital poles, and inferior occipital gyri in the visual cortices. No other contrasts revealed group differences, and no effects of musicians showing stronger activations were found.

## Discussion

In this study, we aimed at investigating possible associations between music training and the maturation of the mechanisms of set shifting during late adolescence and early adulthood. Behavioral performance, P3b responses to targets, and BOLD activations were obtained in a task for set shifting in musically trained and untrained adolescents aged 13–21 years. The results reveal that the musically trained and untrained individuals showed no differences in their performance in the set-shifting task conducted during EEG or during the fMRI. Results from analyses including behavioral test performance from previous measurement rounds showed that the group differences in the neuropsychological set-shifting task diminished with age.

Irrespective of music training, age had an effect on performance in the neuropsychological set-shifting task, with completion time becoming faster with increasing age. This finding is in line with previous research on the development of executive functions showing improvement in performance in executive function tasks until early adulthood (Blakemore and Choudhury 2006; Best and Miller 2010). This enhancement of performance may stem from the maturation of brain structures important for executive functioning (Best and Miller 2010).

Differences in performance in the neuropsychological set-shifting task between the musically trained and untrained individuals were found on previous measurement rounds. In the study by Saarikivi et al. (2016), the then 9–15-year-old musically trained children exhibited better performance than untrained peers in the set-shifting task. In the study by Putkinen et al. (2021), with the participants aged 10–17, the difference persisted. These differences were not explained by better general cognitive abilities or background variables, because the music and control groups were matched in IQ and SES. In line with this, the current results on test performance pooled together from the 3 measurement rounds, with participants aged 9–21, show an overall group difference. However, in performance in the EEG and the fMRI version of the set-shifting task, no group differences emerged. The same was true when only investigating performance differences in the neuropsychological set-shifting task from the last measurement round, with participants aged 13–21. This would suggest that the group differences in the test performance diminished with age. In other words, the music group outperformed the control group in the set-shifting task at younger ages, but the group difference disappeared as the cohort aged.

The results suggest that music training is associated with an advantage in set shifting during childhood but that this advantage does not persist into adolescence and early adulthood. If this were the case, we should not be able to see enhanced set-shifting skills in musically trained adults. Indeed, some studies have not found an association between musical experience or aptitude and set shifting in adults (Bialystok and DePape 2009; Slevc et al. 2016). In the study by Slevc et al. (2016), executive functions were assessed with a wide array of tasks in both the visual and auditory domains. Inhibition was investigated with the Simon task and set shifting with a task where participants alternated between categorizing numbers and letters based on the side of the screen they appeared on. Musical ability or the amount of training did not predict performance in either test but were connected to working memory performance.

Some studies, however, have. In the study by Moradzadeh et al. (2015), switching was investigated with the Quantity/Identity task. Here, the respondent is asked to indicate the identity of digits (the sum of digits presented, e.g. 1 3 = 4) or quantity of digits (1 3 = 2) presented on a screen, or switch between indicating one or the other according to a prompt. In this study, musically trained individuals performed better than untrained individuals, indicating more efficient set shifting.

Our results are more in line with those Slevc et al. (2016) and Bialystok and DePape (2009) who found no differences in performance between musically trained and untrained adults in inhibition and set-shifting ability, respectively. The differences between our results and those of Moradzadeh et al. (2015) may in part be explained by differences in the task used to measure set shifting. The tasks used in studies reporting no differences in set shifting are arguably simpler. For instance, the trail-making task used by Bialystok and DePape requires participants to switch between connecting digits and letters in alphabetical and numerical order. In the task used by Moradzeh and colleagues, participants were required to manipulate the presented information by performing mental arithmetic. It is possible that with such more complex set-shifting tasks requiring more processing of the stimuli, differences between musically trained and untrained participants might still emerge in adulthood.

In contrast with behavioral results, the neuroimaging results indicate slight differences in brain activity during the set-shifting task. No significant amplitude differences at any single channel were observed, but the scalp distribution of the P3b response to incongruent trials was more posterior in musically trained than untrained participants. The P3b is thought to reflect neural mechanisms associated with working memory categorization and updating of mental models (Polich 2003). Adult studies show a posterior distribution for P3b responses (Volpe et al. 2007; Wronka et al. 2012) and therefore the difference in scalp topographies between musically trained and untrained participants signal a more adult-like P3b response in the music group compared to the control group. The result may indicate that musically trained participants reached maturity of updating and working memory functions related to target processing earlier than the musically untrained participants. The difference in maturity no longer provided any benefit in performance in a relatively simple set-shifting task but could still be seen in functional differences related to target processing.

The P3b amplitude correlated negatively with RTs to both congruent and incongruent stimuli in the Arrows EEG task, i.e. the shorter the RT, the larger the response. P3b response amplitudes have previously been found to correlate with RTs (Provost et al. 2018). However, there is no consensus in literature on what exactly the P3b response measured in active paradigms signals, with explanations such as context updating process (Donchin 1981), response selection (Verleger et al. 2005), and stimulus evaluation and response selection (Pritchard et al. 1999). Our findings point towards a link between P3b amplitude and faster processing of targets during a set-shifting task.

Our fMRI results indicated in turn that the music group engaged areas of the dorsal attention network less than the control group on the incongruent trials of the set-shifting task. In addition, visual areas showed less activity, most probably also reflecting less need for cognitive effort in a visual task. Thus, despite the lack of group differences at the behavioral level, individuals in the music group were able to perform the task with more efficient usage of neural resources, signaled by less activation in areas required by the task.

These results are in line with the assertion that expert performance requires less neural resources than that of novices, which has a long history in cognitive neuroscience (Neubauer and Fink 2009). In the domain of executive functions, this “neural efficiency” hypothesis is supported by studies that have linked lower brain activity with experience-related enhancement in inhibition, set shifting, and working memory (Jansma et al. 2001; Sayala et al. 2006; Manuel et al. 2010, 2013; Rodríguez-Pujadas et al. 2014). Moreover, some studies indicate that maturation of executive functions is accompanied by lower brain activity (Luna et al. 2010), while aging has been associated with stronger recruitment of frontal regions in executive functions tasks (Gold et al. 2013). It is noteworthy that the effect in this study was observed for a non-musical visual task, suggesting a domain-general enhancement of the neural systems supporting executive functions in the musically trained adolescents. Furthermore, because the groups were matched in terms of behavioral performance, this effect cannot be attributed to processes like error monitoring but might reflect reduced need for effortful, top-down control in the music group.

The group differences in brain activity were found for the incongruent trials of the set-shifting condition when contrasted with the inhibition condition, as well as with the naming and inhibition conditions combined. Thus, the comparison contrast was between conditions that were identical in terms of stimuli and stimulus–response mapping but differed in cognitive demands. Like many complex executive functions tasks, the incongruent trials of the set-shifting condition tapped into multiple subcomponents of executive functions. In addition to set shifting, these trials required inhibition of the congruent response while the condition as a whole required maintenance of 2 response–stimulus mappings in working memory. The finding that these trials recruited areas of the dorsal attention network in the control group concurs with previous studies that have found activity in these regions in inhibition tasks that require working memory updating, and task switching (Ravizza and Carter 2008; Simmonds et al. 2008; Kim et al. 2012; Lemire-Rodger et al. 2019).

## Limitations

According to many authorities of the field, any study investigating the effects of a formal or informal training (e.g. language or music lessons or via a computer game) should be conducted in a randomized controlled trial study design (Sala and Gobet 2017, 2020). In this design, the participants are randomly allocated to a training or intervention program, thus ignoring their preference or motivation regarding it. In the current longitudinal study, we adopted what we consider an ecologically more valid approach and built the longitudinal study around the participants who were committed to music training since age seven. By this arrangement, we were able to follow up the neurocognitive neural development of the participants for 10 years as revealed by the mean age of the participants of this paper which was 17 years. This could not have been achieved if the participants were given their hobby based on randomization since even programs of 1 year with RCT design suffer from participant drop-out (Tervaniemi et al. 2021). Admittedly, our study outcomes therefore reveal not only an outcome of music training. Instead, possible effects of music training are mixed with factors that lead a child to choose and persist in the musical hobby, such as support from the family and personality factors (Corrigall et al. 2013; Corrigall and Schellenberg 2015). However, in our view, since this is the best approach when investigating the long-term effects of any training as it occurs in real life, such a confound is not to be avoided but to be accepted as part of the phenomenon we tackle when investigating the neurocognitive effects of any type of training spread over many years.

The differences between the neuropsychological and the EEG and fMRI versions of the set-shifting tasks complicates our ability to draw conclusions on group differences. The difference in trial durations, response styles, and the measures of performance between the neuropsychological, original set-shifting task and its EEG and fMRI versions may also explain why no differences in performance between musically trained and untrained participants emerged in the latter 2. The original version of the set-shifting task asks the participant to verbally respond to the stimuli and proceed in a self-paced manner, but as quickly as possible. In the EEG and fMRI versions, in turn, the response happens via a button press, and the stimulus presentation rate is constant, and slower than in the original version. Both the trial length and response style in EEG and fMRI versions may have made these versions of the task less prone to elicit mistakes, and therefore less sensitive in bringing out differences in performance. Also, it is possible self-paced responding gives more information about processing speed and thereby the efficiency of set shifting than RTs, making the neuropsychological version better at bringing out differences in performance. Irrespective of the differences between the tasks used in this study, we feel they still measure the same set-shifting function, as all tasks require changing responses according to rules and stimulus characteristics. Furthermore, musically trained individuals are not more or less accustomed than their untrained peers in responding verbally or pressing buttons during tasks. This means that the presence of lack of group differences is most probably not explained by differences in how the tasks were administered.

## Summary and conclusions

The literature is somewhat mixed with regard to which subcomponents of executive functions differentiate musicians and non-musicians, at which age these differences are seen, and whether they are accompanied by a decrease or increase in brain activity. Our behavioral results from a neuropsychological test for set shifting indicated that during school-age and early adolescence the music group outperformed the control group, but that this group difference had diminished by late adolescence and early adulthood. One explanation for this finding is that during these “cognitive prime years” when executive functions reach full maturity, the musician advantage disappears or requires more difficult tasks to be detected (Moradzadeh et al. 2015). The fMRI results showed that the music group recruited areas of the dorsal attention network less strongly than the control group. This suggests that—despite the lack of group differences at the behavioral level—the musically trained adolescents needed fewer resources to successfully complete the task. Moreover, musically trained adolescents exhibited a more adult-like scalp topography of the P3b response during target processing in the set-shifting task than musically untrained peers. These neural differences were evident in a visual and completely non-musical task suggesting that the enhancement of executive functions in the music group was domain-general.

In sum, our multimethodological results elucidate the neural underpinnings of individual differences in executive functions and are in line with the notion that music training might benefit the maturation of these functions in childhood and early adolescence.

## CRediT authors statement

Katri Saarikivi (Conceptualization, Data curation, Formal analysis, Investigation, Methodology, Visualization, Writing—original draft, Writing—review & editing); Vanessa Chan (Data curation, Formal analysis, Investigation, Writing—original draft, Writing—review & editing); Minna Huotilainen (Conceptualization, Funding acquisition, Project administration, Resources, Supervision); Mari Tervaniemi (Conceptualization, Funding acquisition, Project administration, Supervision, Writing—original draft, Writing—review and editing); Vesa Putkinen (Conceptualization, Data curation, Formal analysis, Investigation, Methodology, Project administration, Software, Supervision, Visualization, Writing—original draft, Writing—review and editing)

## Data availability

The data are available from the corresponding author on reasonable request.

## Funding

This study was funded by the Academy of Finland and the Jenny and Antti Wihuri Foundation.

*Conflict of interest statement*: The authors declare no conflicts of interest.
